# Polygenic risk-stratified screening for cancer: Responsibilization in public health genomics

**DOI:** 10.1177/0306312719858404

**Published:** 2019-06-24

**Authors:** Anne Kerr, Tineke Broer, Emily Ross, Sarah Cunningham Burley

**Affiliations:** School of Sociology and Social Policy, University of Leeds, Leeds, UK; Tilburg Institute for Law, Technology, and Society (TILT), Tilburg University, Tilburg, The Netherlands; Usher Institute of Population Health Sciences and Informatics, Edinburgh Medical School, The University of Edinburgh, Edinburgh, UK

**Keywords:** genomics, responsibility, risk, screening, stratification

## Abstract

In this article, we examine professional discourse around the development of polygenic risk-stratified screening (PRSS) for cancer. Analyzing a range of contemporary professional literatures from Europe, North America and Australia, we explore how the drive to screen for molecular markers of cancer risk makes professionals, screening recipients and publics responsible, in different ways, for acquiring, curating and analyzing molecular data. Investigating how these responsibilities are invoked in discussions of new data practices, technologies, organizational arrangements, engagement, education and protocols for participation, we argue that agendas for PRSS for cancer are both expanding and stratifying responsibilities. Data collection is to be achieved by intensified responsibilities for including, reassuring and recruiting populations, as well as by opening and enriching the datasets on which models and preventative screening arrangements are based. Enhanced responsibilities for screening recipients and publics are also invoked, not just in relation to personal health but for population health more generally, via research participation and consenting to data re-use in the public interest. Professionals, screening recipients and publics are also to become responsible for moderating expectations of screening according to genomic designations. Together these discourses go beyond individual risk management to extend and diversify the responsibilities of practitioners, screening recipients and publics as public health genomics develops.

## Introduction

Genomic research and techniques are key to personalized or precision medicine for a growing range of conditions, notably cancers (Green and Guyer, 2011; Guttmacher and Collins, 2002). In this paper we explore how professional, screening recipient and public responsibilities are being reconfigured as public health genomics evolves.

As England’s Chief Medical Officer’s latest Annual Report, Generation Genome (Davies, 2017: 1) notes, delivering ‘the genomic dream’ in the National Health Service (NHS) requires extensive public engagement and a ‘new social contract between patients and the NHS’. Key here is the move away from what one of the report’s authors frames as ‘paternalistic’ state-based interventions at a population level, to individual responsibility for prevention based on personalized genomic information (Zimmern, 2017). This move would redistribute responsibilities amongst users of population-level services according to their polygenic risk, in order to deliver efficiencies and overcome problems of overdiagnosis in state-based screening programmes.^1^ To achieve this, proponents of public health genomics point to a range of organizational, social and ethical challenges that must be overcome, including public and professional wariness of polygenic risk-stratified screening (PRSS) and associated interventions in health and lifestyle. This invokes a new kind of genomic citizenship (Rose, 2009) with respect to sharing, knowing and tending to genomic risks. Professionals, screening recipients and publics also gain responsibilities for enabling and supporting PRSS, beyond individuals’ responsibility for self-management of polygenic risk. These processes of responsibilization reconfigure the roles and identities of all of these actors as they assume, or are expected to assume, responsibilities for delivering and accommodating new screening arrangements (Boltanski and Chiapello, 2006; Shamir, 2008).

To understand how these processes of responsibilization operate, we conducted a case study of a recent set of professional accounts of research, plans and proposals for PRSS for cancer. Drawing on STS and related social scientific literatures, we widen our analysis from the heightening of individual responsibilities for polygenic risk management to explore the reconfiguration of professional and public responsibilities that sit alongside these processes. We explore the kinds of responsibilities invoked for PRSS research infrastructures, data governance, education and participation, and examine how these are stratified between and amongst professionals, screening recipients and publics. We argue that this diversification and extension of the responsibilities of professionals, screening recipients and publics are important aspects of developing markets in public health genomics.

## Background

STS and other scholars have long been concerned with the implications of genetics and, more recently, genomic medicine for citizenship, particularly the extent to which molecular information might be a resource for determinism and discrimination against particular groups as responsible for their ‘faulty genes’ (Duster, 2015; Lippman, 1991; Shostak and Moinester, 2015). However, there has been little evidence of large-scale discrimination against a genetic underclass (Weiner et al., 2017). But STS scholars have traced the molecular turn in biomedicine, or ‘the social processes and transformations through which phenomena (diseases, identities, pollution, food, racial/ethnic classifications) are re-defined in terms of their molecular components and described in the language of molecular biology’ (Darling et al., 2016: 51). The environment is decentralized (Navon, 2011) and de-emphasized (Timmermans and Shostak, 2016) in genomic research and related health interventions; the influence of social and environmental factors is often measured via their molecular signatures within the body (Darling et al., 2016). Genomics has developed from the One Gene One Disease (OGOD) paradigm (Conrad, 1999) to encompass an appreciation of multiple variants and uncertainties within a paradigm of malleability (Lappé and Landecker, 2015).

However, the paradigm of malleability can still employ old demographic and epidemiological categories that ‘naturalize difference’ and turn attention away from the social processes through which categories are formed and inequalities perpetuated (Prainsack, 2015; see also Duster, 2015). As Ackerman et al. (2016) stress, quantification is part of an unfolding moral economy where behavioral and social risks are located in the body and responsibilities for their prevention rest with the individual. Shostak and Moinester (2015) show how these responsibilities emerge from a range of new genomic fields, such as nutritional epigenetics, where the environment is being rendered internal to the body and molecular mechanisms become a dominant focus of concern and intervention. Meanwhile the broader social-material environment forms a kind of ‘fuzzy background’ (Shostak and Moinester, 2015: 223; see also Landecker, 2011) and the social institutions that negatively affect people’s health and wellbeing ‘fade from view’ (p. 225). Lappé’s (2016) research on how the maternal body is constituted as an environment in autism research makes a similar point, exploring how, as this environment is molecularized, risk is individualized and maternal responsibilities for preventing autism are intensified (see also Ackerman et al., 2016; Arribas-Ayllon, 2016; Darling et al., 2016).

A focus on the molecular reconfigures professional practice across bioscience and biomedicine, particularly in relation to data sharing and collaboration. This brings new responsibilities for interdisciplinarity and translational agendas (Cambrosio et al., 2006; Shostak, 2007) as well as engagement with ethical and political questions about who should take responsibility for the health risks that these new paradigms identify (Darling et al., 2016: 58). Professionals develop a range of technical, institutional and engagement strategies as part of the process of molecularizing fields and disciplines (Shostak, 2005). As with other innovative health technologies, genomics also involves a diversity of social responsibilities for professionals in relation to public engagement and responsible innovation, including the need to work with, educate and enable societal actors to develop new technologies and use services (Davies et al., 2014; Glerup and Horst, 2014).

Building on these insights, in this article we explore how the emergent field of public health genomics is configuring the responsibilities of professionals, screening recipients and publics. In the next section, we explain our approach to generating a corpus of literature and the case study of PRSS for cancer.

## The case study

Public health genomics, sometimes called public health genetics, developed in the early 2000’s in North America and Europe (Shostak, 2003). It was defined by the founding director of the US Centre for Disease Control’s Office of Public Health Genomics, as ‘the application of advances in genetics and molecular biotechnology to improve public health and prevent disease’ (Khoury et al., 2000: 5). This field seeks to integrate new molecular knowledge into many of the activities of public health – from screening or surveillance, through the evaluation and implementation of population interventions to the wider practices of health education/promotion. This involves professionals from a range of applied health fields, notably public health, epidemiology (Bauer, 2013) and bioinformatics (Lewis and Bartlett, 2013). A range of professional bodies have been formed (such as The Public Health Genomics Foundation in the UK and the Centre for Disease Control’s Office of Public Health Genomics in the US) and new journals have come on the scene (such as *Public Health Genomics*, established in 2015). As Bauer (2013) notes, the methodologies of fields such as epidemiology are extending to incorporate concerns such as genomic literacy, which she takes as an indicator of change.

The application of genomic knowledge to cancer screening has been one of the main planks of these developments, applying rapidly developing molecular understanding of cancer etiology to the well-developed infrastructures of cancer screening. Funding agencies such as the European Commission and the Centers for Disease Control and Prevention have made significant investment in a range of large-scale interdisciplinary and international collaborative programs (e.g., the Collaborative Oncological Gene-environment Study in Europe). Researchers have advocated a ‘polygenic approach to disease prevention’ (e.g. Pharoah, 2003), studying the continuous distribution of risk in cancer populations and identifying higher- and lower-risk groups.

To explore how responsibilities are being articulated in this subfield of public health genomics, we conducted a case study of professional discourse on PRSS for cancer. We explore discussions about cancer in general and about particular cancers, notably breast and prostate cancer, as represented in the professional literatures and online commentaries we reviewed. The analysis is also informed by an ongoing multi-sited ethnography of contemporary professional, patient and public experiences and approaches to cancer genomic diagnostics, treatment and screening. Whilst not all of the multiple actors and accounts constitutive of this sub-field are available to our analysis, our dataset nevertheless forms an important element of how professionals circulate knowledge and construct meaning therein (Arribas-Ayllon, 2016; Myers, 1992).

We searched the Medline database for English-language articles published between 2012 and 2016, in order to identify articles on PRSS for cancer. This produced 31 articles (see Supplemental Appendix).^2^ We also searched websites and public-facing reports available via internet searches, to capture the wider discursive processes that make up the subfield. This generated a ‘grey’ literature of an additional ten items, including commentary pieces, professional blogs, research reports and magazine articles (see Supplemental Appendix). Applying situational analysis (Clarke, 2005), we performed a thematic analysis of these texts, which we coded using NVivo, undertaking repeated iterative readings of the texts informed by key themes emerging from relevant STS literatures and our wider data collection (interviews and observations). We focus on how authors frame the possibilities, opportunities and challenges of this emergent field, in relation to understanding genetic, genomic and environmental aspects of disease, risk and its management, how existing and prospective forms of population screening are presented, and the kinds of actors and responsibilities this involves.

The articles, blogs and reports we reviewed are authored by professionals from a wide range of applied disciplines and fields, including bioinformatics, oncology, epidemiology, molecular pathology, genetics, genomic medicine, public health and applied health research. Our sample encompasses a range of studies and reviews: Fourteen articles are quantitative or modelling studies, two are systematic reviews, seven are more general review articles (three of which focus on the ethical, legal and social issues of risk-stratified screening), six involve social research (both quantitative and qualitative), and two are personal view articles (see Supplemental Appendix for an overview of all articles per category). Some articles are published in specialist disease-specific or organ-specific journals and are relatively focused, for instance reporting on specific mutations in a certain form of cancer and reflecting on their implications for screening (nine in total). In addition, two articles were published in internal medicine journals, six in general cancer journals such as *Annals of Oncology*, five in genetics journals, two in cancer/epidemiology journals, one in *Mutation Research*, one in *Cancer Prevention*, one in a public health journal, two in *Public Health Genomics*, and two in medical ethics/internet studies journals. The personal view articles are generally associated with one or more of the blogs/reports we included in the sample. Two of the ten blogs identified were hosted by US agencies: the National Cancer Institute, and the Centers for Disease Control and Prevention (both Government funded). One featured on the UK Public Health Genomics Foundation website (PHG Foundation – an independent UK health policy thinktank) as a supplement to their report on Stratified Screening for Cancer (also discussed in this article).

Several authors from the field of public health genomics are prominent in these discussions. Khoury, the founding director of the US Centre for Disease Control’s Office of Public Health Genomics, is the first author on one of the academic articles and an author on three of the blog reports analysed. Members of a group working with or for the UK Public Health Genomics Foundation (Hall, Chowdhury, Dent) contribute to five of the articles as well as two of the blogs/reports included in our analyses. Pashayan, who used to work for the PHG Foundation, also contributes to eight of the articles and two of the blogs.

## Towards polygenic risk-stratified screening

### Transformative technoscience

In the articles we reviewed, PRSS for cancer commands great promise. Authors frequently emphasize the benefits of rapid development of more precise genomic information and its potential for improving health through new approaches to screening. In typically ebullient language, in a blog post for the Centers for Disease Control and Prevention (CDC), Khoury and Richardson (2015) describe it as ‘usher[ing] in a new era of precision prevention for many diseases in the years to come’. Cancer screening features as a key exemplar of how the emergent multidisciplinary agenda for public health genomics could be translated into practice (e.g., Pashayan et al., 2013). The rapid growth of molecular epidemiology of cancer is presented as a key driver of change in this field. For example, in an article about ‘omics’ and personalizing risk measurements in the online magazine *Cancerworld* (published six times a year by the European School of Oncology), Beishon (2017b) notes that in 2007 ‘only a handful of genes’ for breast cancer had been identified from Genome Wide Association Studies, but by 2017 more than 150 variants were identified for breast cancer and prostate cancer. This presents a narrative of ever more detailed and comprehensive sequencing and subsequent actionability. He quotes Pharoah, a professor of clinical epidemiology at Cambridge and a leading figure in the field, who states that EU funding has supported ‘larger and larger studies needed to find things with smaller and smaller effects’. In a ‘personal view’ in the *Lancet Oncology*, Thomas et al. (2015: e303) describe comprehensive models of polygenic risk estimation as ‘fundamental to the transformative power of genomic technologies in cancer genetics’.^3^

The novelty and power of these new approaches is emphasized further through a form of ‘retrospective accounting’ (Arribas-Ayllon, 2016). Attention is drawn to the limits of prior approaches, including the restrictive focus of single gene studies, the emphasis on families affected by ‘organ-specific, single-gene breast and bowel cancer syndromes’ (Thomas et al., 2015: e303), as well as the dubious clinical validity of some single-high-risk-gene tests developed in commercial environments (Dent et al., 2014). Researchers also point to the continuing problem of ‘missing heritability’ in current approaches, i.e., unidentified rare variants with high or medium penetrance and common variants with low penetrance and additive effects. The classification of cancer risk based on these categories, particularly the ‘binary’ between genes with pathogenic effects and those without, is also queried on the basis that there needs to be a greater recognition of the ‘continuous range of biological variation’ (Thomas et al., 2015: e304). Here the restricted focus of clinical cancer genetics measurements is problematized, particularly the focus on rare mutations and only a few cancers (namely breast, ovarian and prostate). As Thomas et al. (2015) put it, the established methodologies and classificatory systems of clinical cancer genetics are under ‘stress’ (p. e305).

Limitations to established methodologies are also highlighted in articles that explore risk stratification for cancer more generally (where polygenic risk is one of a number of components being considered), particularly in relation to the problems of relying on family history. For example, in a systematic review article on colorectal cancer in *Genetics and Medicine*, an international group of scientists and doctors (Henrikson et al., 2015: 711) conclude that, although family history remains a ‘clinically meaningful’ way to identify those at higher risk and has yet to be superseded by polygenic risk assessment, it is nevertheless ‘an imperfect and dynamic measure’, given that comprehensive family history is not always available and sometimes ‘hidden’ in families. They also caution that ‘family history may be under-reported, less well known, or not a primary influence on the excess colorectal cancer burden in African Americans’ (p. 708). Environmental measures of risk are also presented as inadequate or unrefined in other articles, for example, in those considering how to refine risk measurements based on epigenetic information. Pashayan et al. (2016) note:

‘Information collected on environmental exposures via questionnaire or direct measurement is susceptible to recall bias and to inadequate capturing of exposures with short half-lives and of low biological dose. Epigenetic markers when used in lieu could overcome some of these limitations’ (p. 94).

Together, these repertoires position polygenic risk measurement as more comprehensive and better equipped to capture the complexities of family and environmental exposures and to transform the field of public health more generally. Rapid developments of technological capacity to capture and analyse more data with more complex methods and models in order to produce more accurate estimations of risk are part of a narrative of the transformative power of technoscience in general and genomics in particular. This positions contemporary and historic screening practices as problematic and invokes professional responsibilities for their transformation via more comprehensive, subtle and sophisticated polygenic evidence and models. Researchers from a range of scientific and medical backgrounds are being enrolled in the pursuit of a more refined molecular regime of cancer risk calculation that would better capture and predict the risks of a wider range of cancers, individuals and populations.

### Prospective benefits

This vision of transformation also relies on a form of ‘prospective accounting’ (Arribas-Ayllon, 2016) that emphasizes extending ‘the benefits of personalised risk management developed in the single-gene era to the general population’ (Thomas et al., 2015: e306) to supersede ‘one-size-fits all approaches’ (Pashayan et al., 2013). For example, in another CDC blog Khoury (2013) notes the benefits of moving away from screening applied to the ‘average’ person to a program based on subgroups with different levels of risk. In a report on their European Commission-funded study of prevention and screening for breast, ovary and prostate cancer, the PHG Foundation’s Dent et al. (2014) advocate stratification, because this will provide more effective early treatment for those diagnosed, reduce unnecessary surveillance for lower-risk groups and cut healthcare system costs.

Researchers are nevertheless circumspect about the benefits of polygenic risk stratification in existing programmes, anticipating only modest improvements in the accuracy of current age-stratified and family-history-stratified screening e.g. for breast cancer (Dent et al., 2014). Instead, attention is directed towards the benefits of future stratification. For example, in a discussion of the Gail Model (a risk assessment tool combining a range of factors to estimate the likelihood of a woman developing invasive breast cancer), Beishon (2017b) asserts that adding information on polygenic risk and breast density only modestly increases discriminatory power and instead suggests that ‘the impact could be more substantial in stratifying the population into different risk groups’ (Beishon, 2017; see also Pashayan et al., 2016).

Authors also suggest a range of obstacles that will have to be overcome. These principally relate to the need for an evidence base that draws from genetic and other risk information, supported by public acceptability and health system readiness (e.g. the ‘special article’ in *Genetics and Medicine*, by Khoury et al., 2013). Here the responsibilities of professionals extend to encompass education, evaluation and public relations, building on their core responsibility for data collection. The gathering of an array of further supporting evidence and data is framed as fundamental to the prospects for this kind of screening. Researchers in polygenic risk are encouraged to ‘discover’ the significant number of additional genetic and genomic factors that remain to be identified (Litchfield et al., 2015). Here the population is framed as a resource for further genomic data-collection via existing screening programs. The measurability of genomic data is one of its key advantages, according to these advocates. As Beishon (2017b) notes, it is not that these data are, sui generis, ‘superior’, but that they can be measured ‘incredibly accurately’. In their *Genetics in Medicine* article, Khoury et al. (2013) also prioritize the need for empirical data, arguing that this is paramount to the success of PRSS for common diseases and making the point that although it has yet to be established that PRSS is better than the current model, polygenic data are easy to measure at any point in the lifecourse, as it precedes the development of disease.

In these accounts, benefits are largely prospective and depend on professionals delivering more comprehensive and extensive genomic data and analysis. As epidemiologists Fachal and Dunning (2015) explain in their article on Genome Wide Association Studies to support PRSS for breast cancer, this in turn requires ‘even larger cohorts of breast cancer patients, as well as the development of new statistical methods to comprehensively evaluate combinations of variants conferring low to moderate increases in risk of an already complex disease’ (pp. 38–39). Developments therefore hinge on increased participation in research by screening recipients, patients and wider populations. A particular issue arises with respect to ethnicity, as there is a predominance of participants with European ancestry in these studies. Limited (sub)populations create problems for stratifying individuals. Researchers note that these problems arise because researchers sometimes exclude other ethnicities due to ‘limited sample size’ (Joshi et al., 2014: 1025) or restrict their systematic reviews to studies from Europe, North America and Australia (Mavaddat et al., 2015). Because of such limiations, we see calls for more data to be collected; for example, ‘additional studies will be required to develop and validate genetic profiles for other populations, in particular Asian and African populations’ (Mavaddat et al., 2015: 7).

In these various accounts of the transformative potential of PRSS and its prospective benefits, then, researchers from across the fields of epidemiology, molecular pathology, oncology and public health are responsible for collecting and modelling more data, with more biological features from more individuals and (sub)populations, about more cancers. The generation and measurement of data are key to the ambition of research projects and international collaborations but also the overall vision for PRSS. Crucially, molecular data are particularly valued, because of their amenability to measurement, including as proxies for environmental risks. This approach to data is also presented as qualitatively different from previous methods rooted in the more traditional focus on populations of European descent, family history, close genotype-phenotype associations and rare high penetrance genes in clinical genetics: ‘The genetics of cancer risk will shift from a qualitative basis rooted in a binary classification of variation into a more nuanced and quantitative form’ (Thomas et al., 2015: e307).

Alongside optimistic discourses of transformation and prospective benefits, we also find narratives of incrementalism, with new quantitative analyses and tools of prediction added into existing risk measures and screening programmes. Attention is focused on the molecular level and on particular diseases where screening might be most plausible (i.e., breast cancer and other women’s cancers, colorectal and prostate cancer). Researchers also build upon current genetic and age-stratified screening approaches to develop strategies for common diseases more generally. The uncertainties and limitations of current or previous approaches are to be solved by the collection of yet more data, including about populations with non-European ancestry, to develop more refined models. Here we find echoes of Timmermans et al.’s (2017) analysis, whereby uncertainties are positioned as productive of future improvements in understandings of genetic causality. We also see elements of what Reardon (2013) describes with respect to commercial initiatives such as 23andme, where inclusion in the dataset is presented as a route to improved health and attention is drawn away from the social and environmental risks of disease. In these accounts, problematic inaccuracies in environmental and family history information will be overcome with more measurable, molecular information gathering and more analysis. The acknowledged limitations of these new polygenic approaches in terms of their absolute improvement in risk estimations or their ability to resolve problems of overdiagnosis are also rendered less significant via appeals to the wider transformational potential of stratified screening – ever increasing molecular data collection is both the driver for and the product of these initiatives.

## Extending responsibilities for PRSS

Developing PRSS extends researchers’ responsibilities for the richness and openness of molecular data towards the effective implementation of stratified screening. Dent et al. (2013) note this demands ‘substantial organisational effort’ (p. 98), and Beishon (2017a) stresses how difficult it will be to ‘turn around major public health programs that have considerable bureaucracy and investment in certain IT systems and core beliefs’. Authors claim that transforming organizational infrastructures and cultures is key to the delivery of PRSS (Pashayan et al., 2013) and that radical integrations of discovery and intervention are required to make this work (Thomas et al., 2015). They invoke partnership and organizational entrepreneurship to overcome the bureaucracy, boundaries and core beliefs of current state-based programs that limit the acquisition and analysis of data.

### Data infrastructures

A range of new data protocols for enacting these responsibilities, particularly with respect to data sharing and re-use, are envisaged in the articles we reviewed. Authors set out the case for recruiting screening recipients into ongoing research studies, where their data may be used for purposes beyond the provision of a screening result. For example, Chowdhury et al. (2013), write:

Any proposed stratified screening program must consider in detail the relevance of the many concerns about genetic information, taking into account the technologies that will be used, the implications of the data generated, and its subsequent handling. Important issues will include the need for explicit consent for undertaking analysis of DNA; whether the data generated can, or should, be used for other purposes; the possibility of generating incidental findings and how these should be dealt with; whether the information is relevant for family members and, if so, whether and how it would be shared; whether the data would be stored and, if so, with what safeguards; and who might have access, including the individual, their family members, employers, insurance companies, criminal justice agencies, and researchers. All of these issues must be resolved in the program’s design and implementation to the satisfaction of the public, professionals, and policy makers. (p. 427)

This anticipates an array of responsibilities for ‘health professionals and stakeholders’ (p. 430) to develop appropriate protocols for data handling, communication and safeguarding, including in relation to access arrangements with market-based actors such as insurance companies. Here professionals and interested publics are being enrolled in processes of decision-making about appropriate policies: they are asked to take a view on and influence governance mechanisms. Professionals and lay representatives are also asked to adopt and deliver new kinds of ethical data infrastructures. Dent et al. (2014) also suggest that policy makers need to develop detailed plans that attend to the collection, retention and storage of data:

Since genotypic and phenotypic data are retained for several purposes, more robust and comprehensive systems need to be adopted to safeguard data security, and also to provide an infrastructure for dealing with issues such as the need for re-contact, incidental or unsolicited findings or changes in capacity to consent. (p. 26)

Generating and sustaining these data governance and management infrastructures become a part of professionals’ remit and responsibility to ensure the development of PRSS.

### Extending consent

PRSS agendas also reconfigure the role of screening recipients. Authors (e.g. Chowdhury et al., 2013) stress the need for practitioners to preserve autonomy and not overload patients with complex information. They raise concerns about how to communicate incidental findings as knowledge advances and datasets become richer and more comprehensive. For example, Hall et al. (2013) advocate for policies that ‘refine how risk prediction information is fed back to screening participants, their health providers or potentially affected family members’ (p. 288).

One such policy solution, which the articles we reviewed explore, is ‘dynamic consent’ (e.g. Chowdhury, 2013; Dent et al., 2014). This involves participants agreeing to be re-approached to extend their original consent in the context of new uses of their data. Its advocates argue that dynamic consent offers flexibility and the preservation of autonomy (e.g. Dent et al., 2014) and allows for the possibility that the information on which risk assessments are based might change as more data are gathered and analyzed (Chowdhury et al., 2013). Within these arrangements, professionals involved with PRSS would be expected to acquire ‘adequate understanding’ to use the new kinds of algorithms and risk estimation techniques, and to communicate these with confidence (Dent et al., 2013: 20; see also Chowdhury et al., 2013). Dynamic consent also extends responsibilities for screening recipients, as they are expected to engage with informed choices now and in the future as information about risk develops.

Together these discussions, by colleagues from the humanities, social sciences and public health, extend screening professionals’ and recipients’ responsibilities for the generation and sharing of genomic data, and for onward engagement in the analytical process and uptake of results. This is presented as a process of modernization of institutions, systems and beliefs. Authors invoke collaboration, flexibility and dynamism, contrasting these with the boundaries and rigidities of the past.

### Organizational responsibilities

We find parallel contrasts in discussions of organizational change. The articles we reviewed outlined a range of system-related responsibilities in discussions of the multiple kinds of evaluation required to operationalize PRSS. Evaluation practices are, of course, embedded across health care and service arrangements (Mol, 2008; Pollitt et al., 2010; Power, 1999) and are a primary way in which professional responsibilities are exercised. This is reflected in the literature we reviewed; for example, authors suggest that a wide range of data and processes should be subject to evaluation in order to generate robust information about cost and benefits. As Chowdhury et al. (2013) note,

although modelling can provide estimates of benefit and harm, evidence from empirical data is required to decide whether the true benefits of screening outweigh the true harms. For evaluation of the screening elements of the program, benefits in terms of reduction in morbidity and mortality must be weighed against the harms or costs, including complications of clinical investigations, anxiety over abnormal results, overdiagnosis, and even treatment of false-positive results. (p. 426)

Dent et al. (2014) also discuss the importance of quality assurance:

We recommend that providers of risk stratification incorporating a genotypic element should be transparent about the evidence base and quality assurance processes that are used, to ensure that, regardless of provider, the risk assessments that are generated are safe, robust, and evidence-based. (p. 30)

Once again, the collection of data is key to the exercise of accountability, as is co-operation amongst a range of professionals involved in gathering and analysing data and delivering services. Crucially, professionals are asked to provide evidence and assurance to the health ‘marketplace’, to ensure that ‘policy-makers and consumers’ can compare what is being ‘offered’ when making choices about which service to commission or use (Dent et al., 2014). Here the need to not only collaborate with but also facilitate an emerging market is made explicit.

These various agendas for data infrastructures, consent and organizational change map out a range of new and extended responsibilities. For professionals, this involves modernization, flexibility and integration between fields and across time. Data is the primary asset, not just to produce new findings, but to enable evaluation and quality assurance processes that are key to the development of new markets. Screening recipients play a part in these processes, too, as research subjects who provide data and retain responsibilities for deciding on its uses into the future.

## Reimagining publics and participation

We now turn to consider how the responsibilities of professionals and wider publics are framed in accounts of trust, communication and education. Professionals’ social responsibilities (Glerup and Horst, 2014) include education and cultivation of publics, where publics, in turn, become responsible as partners in PRSS development and governance processes as part of an ‘integration rationality’ of co-production.

### Education and mitigation of (non-)participation

Particularly striking in these accounts is their parsing of publics into selective ‘affected publics’ and problematic or ‘partisan publics’ (Braun and Schultz, 2010). A notable set of concerns emerges about lower-risk publics, members of which could be offered less or no screening as it becomes stratified along genomic lines. Here authors note the strong support for established forms of screening. They suggest that this arises, in part, because of public overestimations of the risk of cancer and of the benefits of screening. For example, in an article on UK women’s attitudes to genetically-stratified mammography screening, Meisel et al. (2015) problematize ‘public perceptions of a “right to be screened”’ (p. 238). In this framing, the problem of overdiagnosis becomes an issue of excessive public demand, rather than a feature of the screening programme in question (see also Khoury et al., 2013; Koitsalu et al., 2016; Kukafka et al., 2015). This focuses attention on problematic publics, invoking the responsibility of professionals to provide, and the responsibility of publics to receive, education and reassurance about PRSS.

As seen in our group of articles, members of this problematic or partisan public cling to old notions of population screening and become inappropriately ‘political’. They are no longer identified by molecular models as needing frequent screening, but they may object to reduced screening as a rationing of health care.

Where there is already an established screening programme, such as that for breast cancer, there may be political or public resistance to a reduction of the screening offered to low-risk groups because women have been encouraged for many years to see screening as universally beneficial and may regard this reduction as service rationing. This may be exacerbated as, inevitably with stratified screening, a small group of women assessed as low risk and receiving less intensive or no screening will subsequently develop cancer. This group may feel let down by the screening programme and would need to be very carefully managed. (Dent et al., 2014: 21)

Authors invoke professional responsibilities for managing these potentially problematic groups (of women). The focus is not simply on education as a corrective. Authors suggest other forms of compromise or mitigation that do not diminish existing services, for example, via extending existing screening programs (such as breast screening) to high-risk groups and not reducing such screening for low-risk groups (even though this would be the logical outcome of stratification). This is contrasted with opportunities for the introduction of a more stratified approach in new screening programmes (Chowdhury et al., 2013). In so doing, authors avoid attenuating the sense of responsibility that motivates participation in screening and cultivate a heightened sense of responsibility amongst newly identified, higher-risk, groups.

These authors also engage in some limited discussion about another kind of problematic public that underestimates its ‘affected-ness’ and ignore or actively resist screening, notably ethnic minority groups.

Ethnic minority status was associated with negative attitudes towards attending screening more frequently with a higher genetic risk, although there was no relationship with attitudes towards risk-stratified screening in general, or towards reducing screening frequency for those at lower risk. This suggests that the prospect of more frequent mammography screening may be problematic for some subgroups. Breast screening attendance has historically been lower for women from ethnic minority groups, with notions of privacy and modesty found to be barriers to breast screening participation. Furthermore, cancer fatalism (i.e. the belief that cancer is inevitable) is higher in some ethnic groups, which has also been suggested as a reason to forego breast cancer screening. (Meisel et al., 2015: 240)

This problematic public is perceived as holding inappropriate beliefs about both cancer and risk behavior. While unlikely to attend for screening if identified as ‘affected’, members of this public are not seen as challenging other elements of stratified screening in general, and thus there is a responsibility for professionals to understand this reluctance as cultural.

### Genomic citizenship

The texts we reviewed also address the question of how to communicate with and educate potential screening recipients around the subtleties of risk and the value of reconfigured or new genomic risk-stratified screening for cancer. Authors advocate tailored communication about particular screening programs. For example, to avoid confusing screening recipients, Dent et al. (2013) suggest that the ‘offer of stratified screening … needs to convey the message that the value of screening depends on whether participants are low or high risk’ (p. 21). This is not just a technical exercise, but involves an overt engagement with politics. For example, in an article on a survey of public attitudes to PRSS for breast and prostate cancer in Sweden, a group of psychosocial researchers note:

Even if screening would be shown to be clinically beneficial, uptake of risk-based screening programs would depend on the attitudes in the population. … Public understanding and interest in participating are essential to the success of risk-based screening, because the individuals will have to understand what is being offered and why in order to consent to it. Moreover, acceptability of stratified screening depends on a recognition that this change in screening routines is in the public’s interest. (Koitsalu et al., 2016: 46)

Here, the authors complement distinctions between affected publics with the cultivation of an ethos of public interest: a rational, lower-risk public would be content not to be screened. They place particular emphasis on the education of these low-risk individuals to avoid the perception of rationing:

One group who may require extra resources and attention are ‘low-risk’ individuals who under a risk-stratified approach may no longer be deemed eligible for screening, or may have a less intensive regimen; some of whom may develop cancer. In order to avoid undermining wider trust in health services, effective communication strategies are needed to ensure that those designated as low risk understand that the rationale in their case for withholding or reducing screening is also to optimize the benefits and reduce screening-related risks. In other words, less screening is about risk reduction not rationing health services. (Hall et al., 2013: 289)

These quotes illustrate a general concern amongst professionals from across disciplines to establish the trustworthiness and hence public acceptance of stratified screening, especially for those who may receive less screening as a result of their lower risk status. In contrast with previous times, when communication was focused on encouraging all (or most) individuals to consider themselves at risk and to be screened, this communication strategy includes an explicit focus on lower-risk individuals. Complex messaging around personal risk and the costs and benefits of screening attends to consumer rights but offsets this against the public interest in order to disavow health care rationing as a driver for these changes. Thus responsibilities for developing more stratified messages sit alongside, rather than eclipse, responsibilities for maintaining classic public health messages. There is still a need to convey the importance of continued vigilance, even amongst those designated low risk and hence likely to receive less screening:

Individuals at lower risk will need to be informed that they may still develop cancer, as is the case with screen-ineligible younger individuals who are considered to be at low risk under the current UK system. (Chowdhury et al., 2013: 428)

These accounts stratify publics’ responsibilities based around genomic designations, with lower-risk subgroups responsible for revising down their ideas about entitlement to screening. Reworked notions of social solidarity sit alongside these changes. For example, in their review of social, ethical and legal aspects of cancer risk-stratified screening, Hall et al. (2013) highlight recent research on genetic solidarity, which they define as ‘the collective commitment of individuals to bear costs to help others with a different genotype’ (p. 289). They express concerns that this might be undermined by genetic variant testing, particularly if it is provided privately, irrespective of risk, raising concerns about rationing and ‘distributive justice’ whereby certain marginalized groups might benefit less from these tests because of a lack of access. Chowdhury et al. (2013) also express concerns that stratified screening could be seen as ‘undermining the principles of solidarity and fairness’ (p. 429) of universal programs, prompting further disengagement by already marginalized groups and worsening inequalities. Hall et al. (2013, 2014) suggest a range of solutions to these problems. For example, in a discussion of childhood genotyping as a feature of stratified population screening, they highlight ‘a need for a composite normative framework’ combining ‘population-centric and individualistic approaches’, including ‘less emphasis on the protection of autonomy’ (Hall et al., 2014: 166). They also call for ‘culturally sensitive and appropriate support’ to different groups to ensure inclusivity, suggesting the need to develop an evidence base beyond models which ‘rely almost exclusively on studies of Caucasian populations’ (Hall et al., 2013: 289), echoing the concerns about the populations from which data are derived.

In these accounts, a multidisciplinary group of professionals begin to articulate ethical frameworks and models of provision for PRSS for cancer. Authors invoke a form of genomic citizenship based around distributing resources in the collective, not just the individual, interest. Participants are expected to provide genomic data without the promise of direct personal benefits, even in the form of screening; this frames participants as both research subjects and as potential recipients of results. This model of citizenship brings with it a need to manage public trust and markets, mitigating the potentially negative perception of stratified screening as a process of rationing through education about risks, research and service development.

### Risk-reducing behaviors

A further set of discussions invokes responsibilities of higher-risk individuals to be committed to preventative action. A lack of such commitment is typically presented as a barrier to effective screening to be remedied by better information and support.^4^ For example, the importance of ‘tailoring’ preventative approaches to encourage appropriate ‘risk reducing behaviours’, including ‘lifestyle changes’ is emphasized in a review article on risk prediction and colorectal cancer (Usher-Smith et al., 2015). Pashayan et al. (2016: 94) also stress the ‘reversibility’ of epigenetic changes as an ‘opportunity for cancer prevention strategies’.

However, in other respects, the public’s growing appetite for risk information and personalized approaches to risk reduction is framed as excessive. For example, Meisel et al. (2013) are concerned that participants have too much faith in risk-avoidance advice and are not sufficiently aware of the limited evidence base for some of this (such as that coming from commercial organizations like 23andme). To some extent, authors frame this faith in risk management as a feature of the search for control in Western society, even though that is inconsistent with other beliefs about cancer and fate. Beishon (2017b) quotes a bioethicist, Inez de Beaufort, to underline the complexities of navigating risk information and the dangers of ‘overload’:

People should also feel free to take some risk in their lives, she said, so how far should health services try and intervene? Will there be services to help people after they have been tested? People are now bombarded with risk information and navigating yet more could be hard.

These kinds of accounts frame the popularity of preventative action as good for recruitment to stratified screening, but this becomes problematic when it is based on over-interpretation or over-dependence on too much or too-vague information, with the market implicated in this proliferation. Professionals, screening recipients and wider publics thereby acquire responsibilities for filtering and navigating a complex array of results from state-based services and commercial outlets to realize the benefits of stratified screening. Together they share responsibilities for being educated about risk information and processes which are growing ever more complex. This extends responsibilities from the ‘constant work of modulation of the self in relation to desired forms of life’ (Rose, 2009: 80), to engaging with and parsing complex genomic information in one’s own and the public interest.

Through these accounts, responsibilities for gathering, managing and interpreting information about individual risk extend to managing the risks and benefits of screening more generally, including the production of responsibilities to and of the state and the collective. Within this, publics are constituted as responsible for calibrating their sense of risk against a widening array of data. Yet they are also presented as being ill-equipped to do so, because of a lack of education, poor communication or a partisan commitment to universal screening. Consequently, members of the public are expected to reorient themselves to appreciate the benefits to society of some actors being given less screening, taking on a sense of genomic and health citizenship more broadly. This reframes public concerns around inequality as a problem of misperception and deploys appeals to science, representativeness or the state to downplay the extent to which these concerns will be realized. Expectations about access and demands for choice in relation to screening also have to be carefully managed. Professionals and publics thereby share responsibility for developing a hybrid commitment to classic public health and consumerism, moralizing state and commercial markets with a sense of the public good including state commitment to a high quality, value-for-money service (Shamir, 2008).

## Conclusion

In this analysis, we have looked at accounts of polygenic risk-stratified screening for cancer. As with other areas of genomics, we find a widening range of diseases and populations being rendered measurable through molecular traces and uncertainties are productive of further molecular data collection (Shostak and Moinester, 2015; Timmermans, 2015). This extends the responsibilities of professionals, screening recipients, and higher- and lower-risk populations and the tools and processes through which they are managed, such as consent and data-sharing arrangements.

Recipients or potential recipients of screening become responsible for developing an appropriate sense of their risk status and therefore eligibility for screening. Lower-risk groups acquire responsibity for developing a sense of proportion around their expectations, whilst higher-risk groups are required to be more vigilant. Incorporating individualization of responsibility for managing risk via dynamic consent processes and intensified surveillance for certain at-risk bodies (Lappé, 2016) and improvements in molecular data collection and education as solutions to perceived inequities rather than structural change (Reardon, 2013; Shostak and Moinester, 2015), PRSS for cancer elaborates and modulates responsibilities beyond the mitigation of personal risk (Rose, 2009). Learning, processing and deriving meaning from ever-more complex genomic risk information is required for population benefit as well as recipient’s or potential recipient’s personal interest. This requires publics to be rational about risk, to be apolitical and non-partisan, to refrain from demanding participation without appropriate (genomic) cause and to work together with practitioners in these endeavors: processes of responsibilization that sit alongside ‘the continuous work of the self on the self’ (Rose, 2009: 80) as genomic risk-based screening develops.

For its advocates, PRSS for cancer is not only a matter of accurately identifying individual risk but of stratifying publics according to their perceptions (for example of entitlements or of discrimination) in order that corrective interventions (education, communication) can be developed. Molecular measures and models align with particular kinds of responsibilities for data acquisition, modelling, sharing and interpretation, articulated through various protocols and exercises in engagement. Here, social scientists lead discussion about how to engage recipients and publics and manage the market in the public interest. Professional responsibilities are being extended alongside those of potential screening recipients and publics, as they work across public-private, institutional, national and disciplinary contexts to prioritize data collection and curation. As part of this, professionals must make arrangements for data stewardship and collection via screening, with an eye to public sentiment and concerns. They must be sensitive to a range of different public perspectives and appetites for/against marketization and to plan services accordingly. Notably, this does not extend to key domains of imperceptibility (Murphy, 2006) regarding education and public involvement. There is a lack of attention to the inadequacies of educational interventions to increase the uptake of screening (e.g. Hollands et al., 2016), or the contribution of screening programmes to excessive public trust and anxiety (Armstrong and Eborall, 2012; Howson, 1999). Perhaps most strikingly, we found little discussion of how to tackle non-participation by ethnic and other minority groups that have had negative experiences of data being reused without consent, or of how to manage minority groups’ concerns through new forms of collective research governance. Although PRSS for cancer brings with it enhanced professional responsibilities for engagement with ethics and politics in relation to education, consent and participation, the repertoire of responsibilities being promoted in these accounts take other responsibilities out of the frame.

The vision of the genomic era explored in this case study of PRSS for cancer is not one in which individuals would be subject to enforced surveillance or discrimination on the basis of their genetic profiles, as critics of geneticization have feared. Nevertheless, our analysis suggests that it does intensify responsibilities for the provision, collection and analysis of molecular data and the configuration of market-state arrangements through which this will be delivered. In so doing, responsibilities are stratified amongst potential screening populations in complex and potentially problematic ways. For example, individuals may be asked to get less screening if they are identified as at low molecular risk, even when their social and economic circumstances are associated with high risk. More generally, there is an expectation in much of this literature that publics will take on new responsibilities to educate themselves about the complexities of stratified risk and notions of genetic solidarity, without acknowledgement of the kinds of work this would generate, especially for the socially disadvantaged. There is also very little attention given to the ways in which professionals will incorporate their enhanced responsibilities to maximise molecular data collection in order to deliver the transformative power of technoscience into already intensive workloads, particularly in public health systems beset with funding crises. Together, these discourses go beyond individual risk management to extend and diversity the responsibilities of professionals, screening recipients and publics to co-produce and consume new markets in public health genomics.

## Supplemental Material

Appendix_Table_of_sources_SSS_article – Supplemental material for Polygenic risk-stratified screening for cancer: Responsibilization in public health genomics Supplemental material, Appendix_Table_of_sources_SSS_article for Polygenic risk-stratified screening for cancer: Responsibilization in public health genomics by Anne Kerr, Tineke Broer, Emily Ross and Sarah Cunningham Burley in Social Studies of Science

## Notes

1. Research has shown that breast cancer screening is associated with high rates of overdiagnosis in nearly 1/3 of new cases and screening is not significantly reducing breast cancer mortality (Bleyer and Welch, 2012).

2. There were some articles we could not include in our sample, such as the CMO report on Generation Genome mentioned above or an article by Hood et al. (2015) on actionable risk genes for health and wellness. We limited our search to cancer-specific articles and on a specific timeframe only, so as to be able to work with a manageable yet comprehensive sample of articles.

3. A second line of research is being pursued in relation to epigenetic changes, and to the possibility of identifying proxy measures for raised epigenetic risk that can be combined with other environmental and genetic risk information to further target screening effectively (Dent et al., 2014; Pashayan et al., 2016). Here attention is focused on the power of combining range of molecular measures for risk estimation which is both more comprehensive and accurate than previous approaches

4. There was limited engagement with clinical and social scientific studies exploring the complexities of how individuals respond to and engage with genetic information, including research that shows that genetic information on risk may not impact on behaviour (Hollands et al., 2016), or research that suggests that biographical histories and ‘innate beliefs’ may outweigh information provided by genetic testing (Bancroft et al., 2014).
