# Supplementary material for: Polygenic risk-stratified screening for cancer: Responsibilization in public health genomics
Source: Soc Stud Sci. 2019 Jun 24;49(4):605–26. doi: 10.1177/0306312719858404 (PMC6688132; doi:10.1177/0306312719858404)
Supplement: Appendix_Table_of_sources_SSS_article – Supplemental material for Polygenic risk-stratified screening for cancer: Responsibilization in public health genomics [file Appendix_Table_of_sources_SSS_article.docx]

**Appendix**

**Table of Sources**

|  | **Title** | **Journal and publication date** | **Authors** | **Type of article/main argument** |
| --- | --- | --- | --- | --- |
| ***Peer-reviewed articles*** | | | | |
| **Empirical/Modelling studies (sorted by cancer/focus article)** | | | | |
| 1 | Public health genomics and personalized prevention: lessons from the COGS project. | Journal of Internal Medicine. 274(5):451-6, **2013** Nov. | Pashayan N; Hall A; Chowdhury S; Dent T; Pharoah PD; Burton H. | A model study looking at risk scores for a range of cancers, which seems promising yet they argue there are a lot of organisational/implementation challenges yet to be addressed before this can be implemented |
| 2 | Polygenic susceptibility to testicular cancer: implications for personalised health care. | British Journal of Cancer. 113(10):1512-8, **2015** Nov 17. | Litchfield K; Mitchell JS; Shipley J; Huddart R; Rajpert-De Meyts E; Skakkebaek NE; Houlston RS; Turnbull C. | Modelling the efficiency of a personalised screening approach for testicular cancer  While this model is generally good at predicting risk, the nature of testicular cancer at the moment does not suggest using personalised screening would be beneficial, however, the authors claim, future advances in genomics might change this |
| 3 | Reducing overdiagnosis by polygenic risk-stratified screening: findings from the Finnish section of the ERSPC. | British Journal of Cancer. 113(7):1086-93, **2015** Sep 29. | Pashayan N; Pharoah PD; Schleutker J; Talala K; Tammela TLj; Maattanen L; Harrington P; Tyrer J; Eeles R; Duffy SW; Auvinen A. | They calculated a polygenic risk score for prostate cancer and then predicted the likelihood of overdiagnosis were this risk score to be used in population screening – in a Finnish population  Conclusion: “Targeting screening to men at higher polygenic risk could reduce the proportion of cancers overdiagnosed.” |
| 4 | Prostate cancer screening using risk stratification based on a multi-state model of genetic variants. | Prostate. 75(8):825-35, **2015** Jun. | Yen AM; Auvinen A; Schleutker J; Wu YY; Fann JC; Tammela T; Chen SL; Chiu SY; Chen HH. | They developed a model to stratify the Finnish population by different risk profiles related to genetic variants to optimize the screening policy, for prostate cancer  The model does discriminate between people more and less likely to benefit from screening |
| 5 | Implications of polygenic risk-stratified screening for prostate cancer on overdiagnosis. | Genetics in Medicine. 17(10):789-95, **2015** Oct. | Pashayan N; Duffy SW; Neal DE; Hamdy FC; Donovan JL; Martin RM; Harrington P; Benlloch S; Amin Al Olama A; Shah M; Kote-Jarai Z; Easton DF; Eeles R; Pharoah PD. | This study aimed to quantify the probability of overdiagnosis of prostate cancer by polygenic risk, and they conclude that polygenic risk could reduce the problem of overdiagnosis  Use of UK-population |
| 6 | Risk Analysis of Prostate Cancer in PRACTICAL, a Multinational Consortium, Using 25 Known Prostate Cancer Susceptibility Loci. | Cancer Epidemiology, Biomarkers & Prevention. 24(7):1121-9, **2015** Jul. | Amin Al Olama A et al | Modelling a polygenic risk score for prostate cancer  Conclusion: Risk proﬁling can identify men at substantially increased or reduced risk of prostate cancer. The effect size, measured by OR per unit PRS, was higher in men at younger ages and in men with family history of prostate cancer. Incorpo- rating additional newly identiﬁed loci into a PRS should improve the predictive value of risk proﬁles. |
| 7 | A genetic score can identify men at high risk for prostate cancer among men with prostate-specific antigen of 1-3 ng/ml. | European Urology. 65(6):1184-90, **2014** Jun. | Nordstrom T; Aly M; Eklund M; Egevad L; Gronberg H. | Study aimed to evaluate whether a genetic risk score identiﬁes men in the PSA range of 1–3 ng/ml who are at higher risk for PCa.  Conclusion: “A risk score based on SNPs predicts biopsy outcome in previously unbiopsied men with PSA 1–3 ng/ml. Introducing a genetic-based risk stratiﬁcation tool can increase the proportion of men being classiﬁed in line with their true risk of PCa.” |
| 8 | A population-based assessment of germline HOXB13 G84E mutation and prostate cancer risk. | European Urology. 65(1):169-76, **2014** Jan. | Karlsson R; Aly M; Clements M; Zheng L; Adolfsson J; Xu J; Gronberg H; Wiklund F. | To explore the prevalence and penetrance of the mutation HOXB13 G84E in a general population. This is linked to prostate cancer, but is not often present in the population |
| 9 | Collaborative Modeling of the Benefits and Harms Associated With Different U.S. Breast Cancer Screening Strategies. | Annals of Internal Medicine. 164(4):215-25, **2016** Feb 16. | Mandelblatt JS; Stout NK; Schechter CB; van den Broek JJ; Miglioretti DL; Krapcho M; Trentham-Dietz A; Munoz D; Lee SJ; Berry DA; van Ravesteyn NT; Alagoz O; Kerlikowske K; Tosteson AN; Near AM; Hoeffken A; Chang Y; Heijnsdijk EA; Chisholm G; Huang X; Huang H; Ergun MA; Gangnon R; Sprague BL; Plevritis S; Feuer E; de Koning HJ; Cronin KA. | A modelling study looking at risk of breast cancer and the frequency of screening most beneficial per risk/age group  They conclude: “Biennial screening for breast cancer is efﬁcient for average-risk populations. Decisions about starting ages and intervals will depend on population characteristics and the decision makers' weight given to the harms and beneﬁts of screening.” |
| 10 | Prediction of breast cancer risk based on profiling with common genetic variants. | Journal of the National Cancer Institute. 107(5), **2015** May. | Mavaddat N. et al | Empirical study: We investigated the value of using 77 breast cancer-associated single nucleotide polymorphisms (SNPs) for risk stratiﬁcation, in a study of 33 673 breast cancer cases and 33 381 control women of European origin.  Conclusion: The PRS stratiﬁes breast cancer risk in women both with and without a family history of breast cancer. The observed level of risk discrimination could inform targeted screening and prevention strategies. Further discrimination may be achievable through combining the PRS with lifestyle/environmental factors, although these were not considered in this report. |
| 11 | Oligogenic germline mutations identified in early non-smokers lung adenocarcinoma patients. | Lung Cancer. 85(2):168-74, **2014** Aug. | Renieri A; Mencarelli MA; Cetta F; Baldassarri M; Mari F; Furini S; Piu P; Ariani F; Dragani TA; Frullanti E. | “This study identiﬁes for the ﬁrst time in non-smokers with lung adenocarcinoma speciﬁc sets of germline mutations that, together, may predispose to this tumor.” |
| 12 | Additive interactions between susceptibility single-nucleotide polymorphisms identified in genome-wide association studies and breast cancer risk factors in the Breast and Prostate Cancer Cohort Consortium. | American Journal of Epidemiology. 180(10):1018-27, **2014** Nov 15. | Joshi AD; Lindstrom S; Husing A; Barrdahl M; VanderWeele TJ; Campa D; Canzian F; Gaudet MM; Figueroa JD; Baglietto L; Berg CD; Buring JE; Chanock SJ; Chirlaque MD; Diver WR; Dossus L; Giles GG; Haiman CA; Hankinson SE; Henderson BE; Hoover RN; Hunter DJ; Isaacs C; Kaaks R; Kolonel LN; Krogh V; Le Marchand L; Lee IM; Lund E; McCarty CA; Overvad K; Peeters PH; Riboli E; Schumacher F; Severi G; Stram DO; Sund M; Thun MJ; Travis RC; Trichopoulos D; Willett WC; Zhang S; Ziegler RG; Kraft P; Breast and Prostate Cancer Cohort Consortium (BPC3). | This seems to investigate interactions between polygenic scores and breast cancer risk factors such as BMI. |
| 13 | Commentary on "common genetic polymorphisms modify the effect of smoking on absolute risk of bladder cancer." | Urologic Oncology. 32(2):213-4, **2014** Feb | Garcia-Closas M et al | Interaction of genetic risk factors with the effect of smoking (cessation) for bladder cancer |
| 14 | Oxidative balance and colon and rectal cancer: interaction of lifestyle factors and genes. | Mutation Research. 734(1-2):30-40, **2012** Jun 01. | Slattery ML; Lundgreen A; Welbourn B; Wolff RK; Corcoran C. | Interaction between genetic and lifestyle factors for colon and rectal cancer |
| **Systematic reviews** | | | | |
| 15 | Risk Prediction Models for Colorectal Cancer: A Systematic Review. [Review] | Cancer Prevention Research. 9(1):13-26, **2016** Jan. | Usher-Smith JA; Walter FM; Emery JD; Win AK; Griffin SJ. | Systematic review of published models (or validation of models) for risk-stratified screening for primary colorectal cancer for asymptomatic individuals  52 risk models were included, most of which showed good discrimination |
| 16 | Family history and the natural history of colorectal cancer: systematic review. [Review] | Genetics in Medicine. 17(9):702-12, 2015 Sep. | Henrikson NB; Webber EM; Goddard KA; Scrol A; Piper M; Williams MS; Zallen DT; Calonge N; Ganiats TG; Janssens AC; Zauber A; Lansdorp-Vogelaar I; van Ballegooijen M; Whitlock EP. | A systematic review to estimate the impact of family history on the natural history of CRC and adherence to screening.  Adherence for colonoscopy higher in people with family history  Conclusion: “Stratiﬁcation based on polygenic and/or multifactorial risk assessment may mature to the point of displacing family history– based approaches, but for the foreseeable future, family history may remain a valuable clinical tool for identifying individuals at increased risk for CRC.” |
| **Review article** | | | | |
| 17 | From candidate gene studies to GWAS and post-GWAS analyses in breast cancer. [Review] | Current Opinion in Genetics & Development. 30:32-41, **2015** Feb. | Fachal L; Dunning AM. | Seems a literature review study looking at the loci currently identified as risk factors for breast cancer, and the future directions in terms of genomic studies looking at breast cancer risk |
| 18 | Genetics of breast cancer: a topic in evolution. [Review] | Annals of Oncology. 26(7):1291-9, **2015** Jul. | Shiovitz S; Korde LA. | Literature review: “This paper will review the known genetic causes of breast cancer and discuss the issues associated with characterizing and under- standing hereditary predispositions to breast cancer.”  Most/all of the high penetrance genes for breast cancer have been identified, and with the advent of genomic techniques lower penetrance genes might be identified too, but this isn’t nor shouldn’t be routine practice yet |
| 19 | Common variants identified in genome-wide association studies of testicular germ cell tumour: an update, biological insights and clinical application. [Review] | Andrology. 3(1):34-46, **2015** Jan. | Litchfield K; Shipley J; Turnbull C. | A review of mechanisms underlying testicular cancer, and an empirical study which used polygenic risk scores to calculate the combined effect of all risk loci on overall TGCT risk and discuss how a potential screening strategy may ﬁt within a broader clinical context. |
| 20 | Can the breast screening appointment be used to provide risk assessment and prevention advice? | Breast Cancer Research. 17:84, **2015** Jul 09. | Evans DG; Howell A. | This article seems to be partly a review of articles on risk-stratified breast cancer screening and prevention, in particular whether the breast screening appointment is a good moment to provide risk assessment and prevention advice to women, as well as reporting some empirical data from their own study – though they never state their objectives explicitly as far as I can tell, and they don’t include a methods section |
| **Review article (social research)** | | | | |
| 21 | Implementing risk-stratified screening for common cancers: a review of potential ethical, legal and social issues. [Review] | Journal of Public Health. 36(2):285-91, **2014** Jun. | Hall AE; Chowdhury S; Hallowell N; Pashayan N; Dent T; Pharoah P; Burton H. | It seems a review of the literature, though they don’t seem to call it a systematic review  This paper reviews some of the main ethical, legal and social issues (ELSI) raised by the introduction of genotyping into risk-stratiﬁed screening programmes, in terms of Beauchamp and Childress’s four principles of biomedical ethics—respect for autonomy, non-maleﬁcence, beneﬁcence and justice. Two alternative approaches to data collection, storage, communication and consent are used to exemplify the ELSI issues that are likely to be raised.  “Ultimately, the provision of risk-stratiﬁed screening using genotyping raises fundamental questions about respective roles of individuals, healthcare providers and the state in organizing or mandating such programmes, and the principles, which underpin their provision, particularly the requirement for distributive justice.” |
| 22 | Stratified cancer screening: the practicalities of implementation. | Public Health Genomics. 16(3):94-9, **2013**. | Dent T; Jbilou J; Rafi I; Segnan N; Tornberg S; Chowdhury S; Hall A; Lyratzopoulos G; Eeles R; Eccles D; Hallowell N; Pashayan N; Pharoah P; Burton H. | A review of the implementation and organisational challenges of risk-stratified screening for a number of cancers |
| 23 | What ethical and legal principles should guide the genotyping of children as part of a personalised screening programme for common cancer? | Journal of Medical Ethics. 40(3):163-7, **2014** Mar. | Hall AE; Chowdhury S; Pashayan N; Hallowell N; Pharoah P; Burton H. | A review of the literature of genotyping young children in order to predict future cancer risk, for a personalised screening programme  “Genotyping neonates, infants or young children as part of a systematic programme would improve coverage and uptake, and facilitate a screening package that maximises potential beneﬁts and minimises harms including overdiagnosis. This paper explores the potential justiﬁcations and risks of genotyping children for genetic variants associated with common cancer development within a personalised screening programme.”  They say that justifications for such a neonate genotyping programme are increasingly compelling |
| **Social Research** | | | | |
| 24 | Poor patient knowledge regarding family history of colon polyps: implications for the feasibility of stratified screening recommendations. | Gastrointestinal Endoscopy. 75(3):598-603, **2012** Mar. | Elias PS; Romagnuolo J; Hoffman B. | An empirical study looking at the knowledge patients undergoing colonoscopy have of family history, which the authors conclude isn’t generally good and therefore family history might not be usable for colorectal screening guidelines |
| 25 | Public interest in and acceptability of the prospect of risk-stratified screening for breast and prostate cancer. | Acta Oncologica. 55(1):45-51, **2016**. | Koitsalu M; Sprangers MA; Eklund M; Czene K; Hall P; Gronberg H; Brandberg Y. | An empirical article examining public interest in and acceptability of risk-stratified screening for breast and prostate cancer  They used a web survey for collecting data – in Sweden  The vast majority was interested in knowing their risk for cancer, and most of them (87%) would agree to more frequent screening but only 27% would agree to less frequent screening |
| 26 | Adjusting the frequency of mammography screening on the basis of genetic risk: Attitudes among women in the UK. | Breast. 24(3):237-41, **2015** Jun. | Meisel SF; Pashayan N; Rahman B; Side L; Fraser L; Gessler S; Lanceley A; Wardle J. | Quantitative study of attitudes among UK women to adjusting frequency of mammography screening based on genetic risk. They conducted home-based interviews with 942 women.  Most women seemed positive about this, and more than half would accept less frequent screening when at lower risk too. Acceptability was less high among ethnic minority women |
| 27 | Why Breast Cancer Risk by the Numbers Is Not Enough: Evaluation of a Decision Aid in Multi-Ethnic, Low-Numerate Women. | Journal of Medical Internet Research. 17(7):e165, **2015** Jul 14. | Kukafka R; Yi H; Xiao T; Thomas P; Aguirre A; Smalletz C; David R; Crew K. | As the implementation of risk-stratified breast cancer screening is complex and, according to the authors, depends on knowledge etc of women, they developed a decision aid programme that would help with understanding of genetic risk and cancer. After using this programme with ‘games’, the authors conducted focus groups with women who spoke English  Conclusion: “In a multi-ethnic population, we demonstrated a significant improvement in accuracy of perceived breast cancer risk after exposure to RealRisks. However, we identified potential barriers that suggest that accurate risk perceptions will not suffice as the sole basis to support informed decision making and the acceptance of risk-appropriate prevention strategies. Findings will inform the iterative design of the RealRisks decision aid.” |
| 28 | Population-based, risk-stratified genetic testing for ovarian cancer risk: a focus group study. | Public Health Genomics. 16(4):184-91, **2013**. | Meisel SF; Side L; Fraser L; Gessler S; Wardle J; Lanceley A. | Focus group study exploring attitudes to the hypothetical scenario of risk-stratified screening for ovarian cancer  They conclude there was a lot of support for this, although they caution that this might be because it is a hypothetical scenario |
| 29 | Incorporating genomics into breast and prostate cancer screening: assessing the implications. | Genetics in Medicine. 15(6):423-32, **2013** Jun. | Chowdhury S; Dent T; Pashayan N; Hall A; Lyratzopoulos G; Hallowell N; Hall P; Pharoah P; Burton H. | “In this article, we summarize the implications of personalized screening for breast and prostate cancers. We report the opinions of multidisciplinary international experts who have explored the scientiﬁc, ethical, and logistical aspects of stratiﬁed screening.” |
| **Personal view** | | | | |
| 30 | How can polygenic inheritance be used in population screening for common diseases? [Review] | Genetics in Medicine. 15(6):437-43, **2013** Jun. | Khoury MJ; Janssens AC; Ransohoff DF. | This seems to be a review/personal view article, although neither is explicitly mentioned. It’s called a ‘special article’ in the journal. It addresses the potential of polygenic inheritance scores for population screening for common diseases among which cancer  They say it has promise but the evidence base is not there yet, with the final sentence: “In the meantime, full engage- ment of the scientiﬁc community, clinical and public health practice, consumers, and policy makers is required to prepare for the evidence-based integration of genomic information into health care and public health practice.” |
| 31 | Clinical implications of genomics for cancer risk genetics. | Lancet Oncology. 16(6):e303-8, **2015** Jun. | Thomas DM; James PA; Ballinger ML. | A personal view on the implications of genomics for cancer risk genetics, both in treatments and in screening/prevention. It seems very much future- rather than present-focused  “In the future, genotype-stratiﬁed public screening and prevention programmes could form part of tailored population risk management. The integration of research with clinical practice will result in so-called discovery cohorts that will help identify clinically signiﬁcant genetic variation.” |
| *Blogs/reports* | | | | |
| 1 | Population screening in the age of personalised medicine | Cancerworld 76 (January/February)**:** 4-11. **2017** | Beishon, M. | Cover story for CancerWorld, looking at what is happening in developments around risk-stratified screening for cancer, with some of the authors of our other articles being interviewed.  It concludes with: “But the signs are that the wheels are turning slowly towards seeing risk-stratiﬁed screening in the prevention ﬁeld – although as well as professional collaboration, there may also have to be much better awareness of the concept of risk among the public.” |
| 2 | 'What can ’omics add to personalised risk assessment?' | Cancerworld **2017** | Beishon, M. | For a disease that has led the field of molecular biology, it is surprising perhaps that so few biomarkers have been identified that can predict a person’s risk of developing cancer. Researchers are now looking at what genetics and epigenetics can add to traditional risk factors such as age, weight and family history, and at how to refine the way we interpret and use the data. Marc Beishon reports.  Includes a discussion on ethics, and on the concept of risk and how complicated a concept this is |
| 3 | 'Could polygenic analysis improve breast-cancer screening?' | The Lancet, commentary, **2002** | Bradbury, J. | A very short (opinion?) article in the Lancet that addresses some of the increased understanding of the genes associated with breast cancer, but that these are not currently sufficient for stratified prevention/screening. However, article written in 2002 |
| 4 | Stratified Screening for Cancer Recommendations and analysis from the COGS project. | **2014** | Dent, T., Chowdhury, S., Pashayan, N., Hall, A., Pharoah, P. & Burton, H. | Report on Collaborative Oncological Gene-environment Study (COGS) (www.cogseu.org) which was an investigation to improve understanding of the causes and prevention of cancer of the breast, ovary and prostate  They seem cautiously optimistic: “Although we do not think the evidence is yet adequate to support risk- stratiﬁed screening, we believe that point will probably be reached before long. Preparing for the change would be wise. In the meantime, further research is needed into impact, utility, cost-eﬀectiveness, acceptability and ethical, legal and social implications. A critical factor may be whether targeting resources according to risk is seen as compatible with the interests of the entire screening population.” |
| 5 | How Can We Use Genetic Testing in Population Screening for Common Diseases? [Online]. | **2013** | Khoury, M. | A blog about scientific and implementation challenges in using genetic testing for population screening  He seems to be quite cautious yet at the same time seems to think risk-stratified screening in the future is very likely: “However, technological developments will drive the interest in using genetic testing for multiple genes to stratify risk in population screening programs and for personal genomic tests.” |
| 6 | Using Genomics in Precision Prevention of Breast Cancer [Online]. | **2015** | Khoury, M. And Lisa Richardson | Online blog on breast cancer risk stratification  “In conclusion, data from two recent large population studies provide more precise estimates of breast cancer risk in women with high risk mutations in BRCA1/2 genes, and in all women based on their polygenic risk profile. These types of studies can influence clinical preventive services with additional evaluation of the utility of this information in reducing the burden of breast cancer morbidity and mortality. In the meantime, [family history](http://www.cdc.gov/genomics/resources/diseases/breast_ovarian_cancer.htm) will continue to serve as a valuable low-tech tool in the stratification of breast cancer risk for screening and prevention. Beyond breast cancer, the large scale epidemiologic investigations of genetic risk will usher in a new era of [precision prevention](http://www.ncbi.nlm.nih.gov/pubmed/?term=rebbeck+precision+prevention) for many diseases in the years to come.” |
| 7 | Evaluating the Clinical Utility of Genomic Variants Derived from Next Generation Sequencing for Opportunistic Disease Screening and Risk Assessment: Evidence Gaps and Priorities. | **2015** | Khoury, M., Cashion, A. & Billings, P. | Seems part of a series: The types of evidence needed to support the use of genome sequencing in the clinic varies by stakeholder and circumstance. In this IOM series, seven individually authored commentaries explore this important issue, discussing the challenges involved in and opportunities for moving clinical sequencing forward appropriately and effectively.  It’s a very brief commentary. Overview of evidence and suggestions for challenges in implementation |
| 8 | Bringing Precision to Screening for Cancer [Online]. | **2015** | National Cancer Institute. | Online blog about a conference held about precision screening. The blog outlines many harms and benefits of screening in general, and situates personalised screening in this context. The need for more evidence is mentioned a couple of times.  "When we’re screening, we’re dealing with otherwise healthy people, and we’re turning them into patients," he said. "We need to do everything we can to [screen people] safely and minimize harms." |
| 9 | 'Integration of genetic and epigenetic markers for risk stratification: opportunities and challenges' | Personalized Medicine 13 (2)**:** 93-95. **2016** | Pashayan, N., Reisel, D. & Widschwendter, M. | An editorial for Personalized Medicine  A brief overview of the literature and what is known about genetic and epigenetic markers, and what is expected to happen in the future  “Cancer is a genetic and epigenetic disease. Combining genetic and epigenetic markers provides huge potentials for risk stratiﬁcation in cancer control programs. Multidisciplinary efforts are needed to overcome the challenges.” |
| 10 | Stratified screening for common cancers [Online]. | **2014** | PHG Foundation. | A very brief text about the COGS again. Not sure if this is a blog or not?  This is their interesting outcome:  “Although the increased risk conferred by each individual genetic variant was low, our findings confirmed (even to sceptics) that the combined information from many variants together provided useful data to refine risk estimates. In the right conditions a more personalised approach to screening, that combines age and polygenetic risk, could increase the number of cancers detected â and prove a cost-effective option.” |
